# Whole Person Modeling: a transdisciplinary approach to mental health research

**DOI:** 10.1007/s44192-023-00041-6

**Published:** 2023-08-24

**Authors:** Daniel Felsky, Alyssa Cannitelli, Jon Pipitone

**Affiliations:** 1 Krembil Centre for Neuroinformatics, Centre for Addiction and Mental Health, 250 College Street, Toronto, ON M5T 1R8 Canada; 2 Department of Psychiatry, Faculty of Medicine, University of Toronto, Toronto, ON Canada; 3 Division of Biostatistics, Dalla Lana School of Public Health, University of Toronto, Toronto, ON Canada; 4 Rotman Research Institute, Baycrest Hospital, Toronto, ON Canada; 5 Faculty of Medicine, McMaster University, Hamilton, ON Canada; 6 Department of Psychiatry, Queen’s University, Kingston, ON Canada

**Keywords:** Whole Person Modeling, Transdiciplinary, Mental health, Psychiatry, Biopsychosocial

## Abstract

The growing global burden of mental illness has prompted calls for innovative research strategies. Theoretical models of mental health include complex contributions of biological, psychosocial, experiential, and other environmental influences. Accordingly, neuropsychiatric research has self-organized into largely isolated disciplines working to decode each individual contribution. However, research directly modeling objective biological measurements in combination with cognitive, psychological, demographic, or other environmental measurements is only now beginning to proliferate. This review aims to (1) to describe the landscape of modern mental health research and current movement towards integrative study, (2) to provide a concrete framework for quantitative integrative research, which we call Whole Person Modeling, (3) to explore existing and emerging techniques and methods used in Whole Person Modeling, and (4) to discuss our observations about the scarcity, potential value, and untested aspects of highly transdisciplinary research in general. Whole Person Modeling studies have the potential to provide a better understanding of multilevel phenomena, deliver more accurate diagnostic and prognostic tests to aid in clinical decision making, and test long standing theoretical models of mental illness. Some current barriers to progress include challenges with interdisciplinary communication and collaboration, systemic cultural barriers to transdisciplinary career paths, technical challenges in model specification, bias, and data harmonization, and gaps in transdisciplinary educational programs. We hope to ease anxiety in the field surrounding the often mysterious and intimidating world of transdisciplinary, data-driven mental health research and provide a useful orientation for students or highly specialized researchers who are new to this area.

## Introduction

### The burden of mental illness

Mental illness is a major global concern receiving significant attention from public health experts, policymakers, and society as a whole. In 2019, the Global Burden of Diseases, Injuries, and Risk Factors Study reported that mental disorders accounted for approximately 1 billion cases across 204 countries and territories—an increase of 48% over the preceding 20 years [1]. At that time, the most prevalent disorders were depressive disorders and anxiety disorders, with age-standardized Disability-Adjusted Life-Years (DALYs; the sum of years of life lost to due to premature mortality and years lived with disability due to illness) for all mental disorders estimated to be 1,566 per 100,000 people (equivalent to a total of 125 million lost years of life globally) [1]. Isolation and socioeconomic instability accompanying the global COVID-19 pandemic has made matters worse: a systematic review and meta-analysis of 107 studies reporting on mental health issues during the pandemic found a global prevalence of 28% for depression, 27% for anxiety, and even higher numbers for stress-related symptoms [2]. Adding insult to injury, several major societal and lifestyle causes of mental illness, especially in youth (e.g. social media use [3], economic stress [4, 5], nicotine vaping [6], and physical inactivity [7–9]) are on the rise.

Projections for global population demographics suggest that increases in life expectancy and decreases in fertility will result in octogenarians outnumbering children under 5 by a ratio of two to one by the year 2100 [10]. As part of this trend, the condition with the greatest proportional increase in serious health-related suffering between 2016 and 2060 is expected to be late-life dementia [11], of which the most common cause is Alzheimer’s disease [12, 13]. Mental Illnesses such as these not only impact the quality of life and well-being of those affected and their families, friends, and caregivers, but also have significant economic implications, as they result in lost productivity, increased healthcare costs, and reduced workforce participation [14].

### Slow progress in treatment

Despite this global problem, progress in our ability to prevent and treat mental illness, or to promote mental wellness, has not kept pace. While a publicly held notion of psychiatry as a failed medical discipline [15, 16] is misrepresentative of the advances that have been made [17], it appears as though scientific investment in other areas of medicine has born more fruit. For example, while cancer remains among the top global causes of death [18], survival rates for most cancers have improved steadily for the last four decades, albeit in high-income countries [19–21]. This is due to many milestones and success stories [22], including improvements in screening methods for early detection [23], the successful deployment of tailored precision treatments [24, 25], and the wide adoption of public policies which have successfully reduced the prevalence of tobacco smoking (the major cause of lung cancer, which accounts for the largest proportion of cancer-related deaths globally [26]) [27]. In addition, data from The Cancer Genome Atlas (TGCA) Project [28] has led directly to the discovery of molecular mechanisms of cancer growth and FDA-approved precision treatment strategies (e.g. Larotrectinib, which specifically targets tumors with *NTRK* gene fusions [29]), which can dramatically improve patient outcomes and side effect profiles [30].

Similar modern breakthroughs in the diagnosis and treatment of mental illness have been absent. The first effective treatments for mental disorders, which involved inducing either a coma or convulsions via insulin-induced hypoglycemia or electric shock (electroconvulsive therapy; ECT), were developed in the 1930s [31, 32]. Despite its brutal and archaic portrayal in popular culture [33], ECT remains the most effective treatment option for otherwise treatment-resistant depression [34, 35]—even being described as “the most effective and rapid-acting long-term somatic treatment in psychiatry” [36, 37]. The first antipsychotic drug, chlorpromazine, was discovered by chance in 1951, originally intended for use as a surgical anesthetic [38]; its mechanism of action, dopamine D2 receptor binding, was not discovered until 1975 [39], and this remains the principal brain target of all antipsychotics prescribed today [40]. The first antidepressant, isoniazid—which inhibits the monoamine oxidase enzyme and is the same mechanism of action for a class of antidepressants still used today [41]—was also discovered serendipitously in 1950 after patients treated for tuberculosis experienced improved mood [42]. Three out of five therapeutics currently available to treat Alzheimer’s disease act to increase brain levels of acetylcholine [43], a non-disease modifying mechanism proposed in the late 1970s [44, 45]. Cognitive-behavioural therapy (CBT), a group of widely adopted and effective non-pharmaceutical interventions [46], was developed in the 1950s and has not changed fundamentally since. Recent developments, such as the use of transcranial magnetic stimulation (TMS) for treatment of major depressive disorder and schizophrenia [47–49], have generated excitement despite the fact that they are not effective for everyone, not widely available, and not mechanistically understood [50].

In the realm of diagnostics, the main tools are the fifth edition of the Diagnostic and Statistical Manual (DSM-V-TR) [51] or International Classification of Diseases 11th Revision (ICD-11) [52] which both define clusters of symptoms fulfilling criteria for discrete diagnoses. This is sometimes referred to as “Kraeplinianism” [53], since the nosological principles on which these classification systems are based were founded by Emil Kraeplin well over 100 years ago. As fundamental diagnostic tools for mental illness, they are widely seen as deeply provisional, and are often criticized as being incoherent, heterogeneous, provincial, and unrelated to causal biology [54, 55]. Furthermore, these diagnostic categories can be misleading: individuals with the same diagnoses may be more dissimilar to each other in symptoms than individuals with different diagnoses [56], and diagnostic error is prevalent [57, 58].

### What is hampering progress in mental health research?

The contrast between the rising burden of mental illness and our lack of available solutions raises the question: why are we having such a difficult time successfully correcting the trend of increasing suffering due to mental illness? The answer to this question will vary wildly depending on who you ask: The geneticist may answer “*we have not yet identified the causal genes and variants for mental illness*”; The biotechnologist may answer “*we have not yet perfected the techniques required for tolerable gene therapy*”; The neuroscientist may answer “*we have not yet identified the causal brain circuits and cells which respond to stimulation*”; The public health specialist may answer “*we have not yet identified the policies responsible for systemic inequity*”. All would agree there are gaps in our understanding of mental illness across multiple research domains, but what to do about it?

A two-part list published in 2015 by a group of internationally-recognized experts in psychiatric research [59, 60] suggested that progress in mental health research can be divided into two sets of deep theoretical and practical problems (inspired loosely by the famous Hilbert problems in mathematics): those related to (a) nosology and diagnostics and those related to (b) etiology and pathophysiology. Jakovljevic and Jakovljevic [61] extend this list to include a third category, (c) achieving stratified, precision treatments, and list as one of the key problems: “How to integrate different but complementary branches, theories, and practices within psychiatry?”. In this paper, we explore the view that progress in mental health research is hampered by the divisions between research disciplines and that prioritization of transdisciplinary data-driven approaches is what is needed.

The goals of this review are: (1) to describe the landscape of modern mental health research and current movement towards integrative study, (2) to provide a concrete framework for quantitative integrative research which we call Whole Person Modeling, (3) to explore existing and emerging techniques and methods used in Whole Person Modeling, and (4) to discuss our observations about the scarcity, potential value, and untested aspects of highly transdisciplinary research in general. We hope to ease anxiety in the field surrounding the often mysterious and intimidating world of transdisciplinary data-driven mental health research and provide a useful orientation for students or highly specialized researchers who are new to this area.

## The organization of mental health research

### Popular theoretical models of mental illness

Understanding mental health and illness requires the development of theoretical models that help explain complex interactions between biological, psychological, and social factors. A recent review of 110 publications identified 34 different models of mental health problems [62] generally falling under the categories of Biology, Psychology, Social, Consumer, and Cultural. Different models often emphasize specific aspects of mental health, such as biological, cognitive, emotional, or social processes.

Broadly, the perspectives of each of these models can be understood in one of two dominant frameworks. In the biomedical model (reviewed by Deacon [63]) mental illness is seen as fully reducible to biological elements. It asserts that mental disorders are caused by biological abnormalities, primarily located in the brain, and therefore emphasizes genetics, neurochemistry, and brain structure. The dopamine hypothesis of schizophrenia [64], which posits the cause as primarily an imbalance of dopamine in the brain, or the serotonin hypothesis of depression [65], which suggests that low levels of serotonin contribute to depressive symptoms, are prime examples of the biomedical paradigm which dominated mid-twentieth century mental health research. In response to what was seen as an overly reductionist approach, the biopsychosocial model was introduced by psychiatrist George Engel in the late 1970s [66]. Engel proposed that mental illness was not fully reducible to biological elements, but instead resulted from a complex interplay of biological, psychological and social/cultural forces. Examples of the biopsychosocial model include the diathesis-stress model of mental illness [67], which posits that mental disorders result from the interaction between an individual's genetic vulnerability (diathesis) and stressors in their environment (stress) or the vulnerability-stress-adaptation model, which emphasizes the role of coping and adaptive processes in the development and maintenance of mental health disorders. The biopsychosocial model is presently the dominant framework in mental health research. Importantly, neither “model” should be viewed as a scientific or philosophical model in the sense that it provides specific explanatory variables or mechanisms. Rather, they simply act as a way to frame discussions about the causes of mental illness [68].

That said, which model one adopts may influence which diseases are seen as a psychiatric illness and which are excluded. In this paper, we present many examples of research on neurocognitive illnesses, such as Alzheimer's and Parkinson’s disease, which have psychiatric manifestations but owing to our greater understanding of the neurobiological basis of these diseases might be seen now as ultimately more neurologic than psychiatric. As we describe below, although the Whole Person Modeling approach mandates inclusion of biological factors when modeling, it does not restrict findings from challenging the assumption of a purely (or even significant) biological basis for a given mental illness. In particular, this approach is compatible with theoretical models such as dynamical symptom networks [69] or computational psychiatry methods [70] that attempt to explain mental disorder at levels of abstraction above the neurobiological.

### Disciplinarity in traditional mental health research

Despite the field’s acceptance that mental illness results from a complex combination of biopsychosocial influences, its research landscape remains largely compartmentalized and siloed. The term disciplinarity refers to this compartmentalized way of thinking and operating. For example, psychiatry has primarily concentrated on clinical manifestations and diagnoses, whereas psychology has delved into the cognitive, emotional, and behavioral underpinnings of mental health disorders. Psychiatric geneticists are concentrated on identifying the genetic variants conferring heritable risk for mental illness while cognitive neuroscientists are dissecting brain circuits involved in disease-relevant thought processes.

This divided structure is reinforced by the major organizations and consortia that represent intellectual leadership in mental health research. For example, the American Psychological Association (APA) is divided into 54 divisions, with only one (“Society for General Psychology”) dedicated to the integration of cross-disciplinary knowledge. The Psychiatric Genomics Consortium (PGC) [71], which has been foundational for bringing together genetic researchers from around the globe and publishing benchmark genome-wide association studies (GWAS) on psychiatric illness, is now divided into 15 working groups, two of which (“cross disorder analyses” workgroup, which has been part of the PGC since 2008, and the “cross-population analyses” special interest group, which was formed in 2018) are explicitly dedicated to multi-trait and multi-population analyses. Universities themselves are highly organized into disciplinary siloes, which may represent a foundational barrier to the propagation of cross-disciplinary thinking. Associated challenges have been articulated by Kendler (2014):

*“…developing integrationist research programs presents both practical and conceptual challenges. Practically, the nature of specialization in scientific psychiatry makes it a challenge to develop excellent cross-disciplinary groups and to obtain the needed research samples and funding”* [72].

### The limits of disciplinarity

The fingerprints of organizational and disciplinary compartmentalization can be found on the research that typifies the field. In an analysis of 197 published articles on the causes of psychiatric disorders, over two-thirds contained an analysis of variables limited to only one level of either the biological, psychological, or environmental [72]. Those studies that did include multi-level analyses were generally limited to only a few disciplines (e.g. neuroscience and neuropsychology, and genetic and environmental risk factors). Although each of these studies represents an attempt at understanding a specific aspect of mental illness, by nature of being restricted to one or few domains of analysis they necessarily overlook the intricate interplay of biopsychosocial factors we know determine mental health. For example, it has been shown that self-reported reasons for depressive episodes are not correlated with objective risk factors (such as early life stressors and family history) for depression [73], suggesting a vast gap in our understanding of the causes of depression that cannot be crossed by approaching from the subjective or objective alone.

The limits of this disciplinary structure are also made apparent by instances where an assumption based on incomplete information is later revealed to be flawed by cross-disciplinary interrogation. One example of such scientific myopia is the heavy reliance on the amyloid hypothesis in the field of Alzheimer’s disease, sometimes criticized for stunting growth of innovation in therapeutics for dementia [74, 75]. This hyperfocus on one molecular mechanism for nearly two decades led to the development and testing of several monoclonal antibody drugs that target the accumulation and aggregation of beta amyloid protein, one of two definitive brain pathologies for Alzheimer’s disease. To date, most phase 3 clinical trials of anti-amyloid drugs have failed to meet primary endpoints [76, 77]. Mixed results have been found in patients who are also imaged with positron emissions tomography (PET) or assessed for existing genetic risk factors for Alzheimer’s disease—a precision treatment approach—but the field remains grappling over new ways to stratify patients for different treatments [78, 79]. Assessing signs of early cognitive decline alone to indicate treatment with amyloid-targeting therapeutics is not enough, and new cross-disciplinary approaches are yielding not only new pharmacological targets [80], but also pilot programs for highly personalized treatment based on science in nutrition, infectious diseases, endocrinology, sleep, physical exercise, and gastroenterology [81].

Indeed, we have seen in the last century a transition from purely psychodynamic-based explanations of mental disorder, to the descriptive psychiatry of neo-Kraeplinism and phenomenological criteria of the DSM-III, to more recent efforts at systematizing integrative modes of research exemplified by the Research Domain Criteria (RDoC) project. Although many classes of mental disorder stubbornly resist complete biological characterization despite great effort—possibly due to overinvestment in single technologies, modalities, or mechanisms [70]—we have seen discoveries from across disciplines transform what were once purely primary psychiatric diagnoses into those understood as medical illness with psychiatric manifestations (or in DSM-5-TR parlance, disorders “due to medical illness”). One set of important discoveries include the recognition of secondary psychotic disorders, such as the autoimmune encephalitities or psychosis related to seizure disorder, which are helping to provide working models for understanding the neurobiological basis of primary psychotic disorders such as schizophrenia [82]. Major depressive disorder secondary to severe hypothyroidism consists of a mental state indistinguishable from depression arising seemingly without cause, yet identification of endocrine deficiencies and treatment with levothyroxine can correct the biochemical deficiency and reverse symptoms [83]. Pheochromocytoma [84], a rare type of adrenal tumor, causes symptoms mimicking the acute anxiety of panic disorder which can be cured entirely by the tumor’s removal.

Historically, reasons for methodological or theoretical fixation could include practical limitations on study design or, more troublingly, enduring dogma [85, 86] (see Sect. 4.5). Under these restrictions, researchers conventionally consider only one or two independent variables, exposures, or predictors at a time. Some study designs—such as double-blind randomized controlled trials—seek to avoid bias caused by heterogeneity (or “noise”) in highly complex phenomena surrounding their central questions (e.g. through experimental or natural randomization [87]), though they are unable to explain it. Depending on a researcher’s area(s) of training, analyses may embrace or minimize complexity, and appropriately so. However, those looking to explore and test the biopsychosocial model of mental illness with data have been historically stymied, as the datasets required—i.e. those spanning multiple modalities of biopsychosocial human data collection across populations and time—did not exist. This has changed within the last decade.

### Toward an era of cross-disciplinary mental health research

In the era of large-scale genomic, neuroimaging, and digital health data collection, institutionally mandated data sharing policies, and accessible, scalable, multivariate computational methods [88], more fulsome analyses that span multiple disciplines are well within reach. Funding organizations, governments, and scientific leaders recognize this, and have been actively supporting and incentivizing integrative research for years.

In 2016, The US National Institutes of Health (NIH) Precision Medicine Initiative (PMI) was launched with an investment of US$215 million toward the development of research into precision medicine [89, 90]. The major outcome of this investment was the establishment of a nationwide study called the All of Us Research Program [91]. In describing the capabilities of this program, the 2015 PMI Working Group Report to the Advisory Committee to the Director of the NIH, states (on page 1):

*“The Working Group identified a number of high-value scientific opportunities or use cases that could be used to inform the design of the PMI cohort. These cases include: development of quantitative estimates of risk for a range of diseases by integrating environmental exposures, genetic factors, and gene-environment interactions.” *[92]

The US National Institutes of Mental Health (NIMH), which funded approximately US$1.5 billion across over 2,000 extramural mental health research grants and contracts in 2022 [93], includes the Division of Translational Research (DTR), which supports:

*“…integrative, multidisciplinary research on the following areas: the phenotypic characterization and risk factors for psychiatric disorders; neurobehavioral mechanisms of psychopathology; trajectories of risk and resilience based on the interactive influences of genetics, brain development, environment, and experience; and design and testing of innovative psychosocial, psychopharmacologic, and somatic treatment interventions.”* [94]

The Wellcome Trust, an independent charity which prioritizes funding for depression, anxiety, and psychosis, and funded £866 million in health and mental health-related research in 2021/2022 [95], lists as their first priority under “What do we want to achieve?” in the mental health funding section of their website:

*“We want to: gain a better understanding of how the brain, body, and environment interact in depression, anxiety, and psychosis so that we can spot potential points for early intervention.” *[96]

A 2001 report by the Division of Health Promotion and Disease Prevention within the Institute of Medicine at the National Academy of Sciences [97, 98] delivered nine specific recommendations for the future of research aimed at promoting health. The first of these was:

*“Understanding psychosocial and biobehavioral mechanisms that influence health is critical to better understand and tailor intervention efforts. Research in this area should be encouraged.” *[97]

The Canadian Institutes of Health Research (CIHR), the principal public funder of health research in Canada, is composed of 13 institutes, but makes explicit their intention of organizing in “virtual”, interdisciplinary structures:

*“Unconstrained by bricks and mortar, the Institute's virtual structure encourages partnership and collaboration across sectors, disciplines and regions.” *[99]

Even further, in their Strategic Plan 2021–2031, CIHR lists as their second of three strategies for pursuing health equity through research:

*“CIHR will develop an action plan that identifies and addresses the social, cultural, environmental, structural, and biological determinants of health…” *[100]

These clear shifts toward promoting integrative, transdisciplinary science have led to the formation of major public–private collaborations and the collection of large-scale human datasets for mental health and other broadly health-related research. Some examples of such datasets include (sample sizes (n) as of 02/2023): FinnGen (n = 589,000) [101], UK Biobank (n = 500,000) [102], All of Us (n = 372,380; target n = 1,000,000) [91], the Million Veterans Program (n = 930,000) [103], the Electronic Medical Records and Genomics Network (eMERGE) network (n = 136,078) [104], and the Canadian Partnership for Tomorrow’s Health (CanPath, composed of seven regional cohorts; total n = 331,359). Datasets collected by for-profit corporations, such as 23andMe [105], are also sometimes used in mental health research to boost sample sizes alongside aggregated and meta-analyzed data from dozens or hundreds of individual component studies (e.g. the international Genetic Investigation of Anthropometric Traits (GIANT) Consortium [106, 107], the Global Boibank Meta-analysis Initiative (GBMI) [108]). While together these studies include several million participants, not all have completed assessments for different types of biopsychosocial measures. For example, out of over half a million UK Biobank participants, only 1/5 are currently being invited for structural and functional neuroimaging with MRI [109] (which is still a remarkable feat, considering that in 2015 the median example size for functional MRI studies was less than 30 [110]).

Other data collection efforts have been designed to prioritize depth of characterization and breadth of data types collected, rather than to maximize recruitment numbers. These studies are often targeted to a specific age range or clinical group, and can include repeated assessments over many years that capture genetic, imaging, and clinical data. Examples of such studies include the Religious Orders Study and Rush Memory and Aging Project (ROS/MAP; n = 3,795) [111] (which is part of the larger US National Institute on Aging (NIA) Accelerating Medicines Partnership® Program for Alzheimer's Disease (AMP-AD)), the Alzheimer’s Disease Neuroimaging Initiative (ADNI; target n = 2000) [112], the Adolescent Brain and Cognitive Development Study (ABCD; n = 11,880) [113], the Dunedin Study (n = 1037) [114], the multigenerational Framingham Heart Study (FHS; n > 15,000) [115], and the Lothian Birth Cohort (n = 70,805) [116] – though we note that several smaller cohorts that comprise larger consortia mentioned above (e.g. the comprehensive cohort of the Canadian Longitudinal Study on Aging (CLSA) [117], part of CanPath) are similarly deeply characterized.

The datasets mentioned above do not constitute an exhaustive list of available research resources. Manageable starting points for exploring, and ultimately accessing, the bewildering range of available datasets for cross-disciplinary analysis include the Maelstrom Catalog (https://www.maelstrom-research.org/) [118] the NIH database of Genotypes and Phenotypes (dbGaP) (https://www.ncbi.nlm.nih.gov/gap/) [119], and the NIMH Data Archive (https://nda.nih.gov/).

### Terminology of integrative, cross-disciplinary research

Wading safely into the waters of data-driven, cross-disciplinary research in this era of “big data” requires effective and precise communication. To study multiple data types, the researcher must either have sufficient expertise with all of them or work within a team or network who do so collaboratively. This is because an adequate understanding of the nuances, interpretations, and limitations surrounding the use of a specific type of data—which falls within the broader term of “domain knowledge” or “domain expertise”—is essential for conducting responsible and useful research. This section will define and contrast a number of commonly used terms and concepts with the aim of orienting the reader to Whole Person Modeling.

#### Cross-disciplinary, multidisciplinary, interdisciplinary, transdisciplinary

Cross-disciplinary, multidisciplinary, interdisciplinary, and transdisciplinary are terms used to describe various approaches to research that involve the collaboration or integration of different disciplines (reviewed by [120]). Sometimes, these terms are referred to under the umbrella of “polydisciplinary” (or pluridisciplinary) approaches, in contrast to within-disciplinary or “intradiscipinary” (or monodisciplinary) approaches [121]. While these terms all imply collaboration and involvement of multiple disciplines, they differ in the manner and extent to which disciplines are combined.

**Cross-disciplinary:** This catch-all term may refer to any polydisciplinary approach [122]. The focus is often borrowing or adapting ideas from one field and applying them to another, without necessarily integrating or synthesizing the disciplines.

**Multidisciplinary (additive):** This approach involves the parallel and independent contributions of different disciplines to a common research objective. In multidisciplinary psychiatric research, experts from various fields might work together on a shared project, but they maintain their disciplinary boundaries and methodologies. The individual findings from within each disciplinary boundary may or may not then be combined.

**Interdisciplinary (interactive)**: Interdisciplinary research involves the collaboration and interaction of experts from different disciplines to tackle a common problem or research question. This approach goes beyond borrowing ideas and seeks to create new knowledge and understanding by integrating multiple perspectives, though this integration may not span the boundaries of natural, social, and health sciences.

**Transdisciplinary (holistic):** Similar to interdisciplinary, transdisciplinary research specifically transcends the boundaries of individual disciplines, aiming to create a unified framework or approach that integrates theories, methodologies, and concepts from multiple disciplines [123]. Transdisciplinarity is different from interdisciplinarity in that it describes the integration of natural, social, and health sciences.

#### Multimodal, multivariate, multilevel, and multi-scale

**Multimodal** research designs involve the combination of multiple data types and methodologies. For example, a study collecting both genetic and neuroimaging data can be considered multi-modal. On the other hand, a study collecting multiple types of only neuroimaging data, such as from T1-weighted, T2-weighted, and diffusion weighted scans, can also be considered multi-modal. In contrast to a unimodal approach, a multi-modal approach has the potential to reveal previously unrecognized connections between data types, or between views or perspectives of the same data type, and may facilitate the development of more complete and accurate models.

**Multivariate** refers to the analysis of multiple variables simultaneously, either by inclusion in a single statistical model or by comparing multiple models, often with the goal of identifying or describing relationships and patterns among them. This approach can clarify the independent and interdependent effects of several factors or phenomena at the same time, potentially providing a deeper understanding of an illness or outcome than a univariate (one variable; e.g. a one-sample t-test of a sample mean against a hypothesized value) or bivariate (two variables; e.g. Pearson correlation) approach. Common multivariate techniques, such as multiple regression, factor analysis, and canonical correlation analysis, are often used to identify meaningful associations and uncover latent structures in complex datasets. Multivariate models can be relatively simple (e.g. multiple linear regression with two predictors, estimating 3 parameters) or incomprehensibly complex (e.g. deep neural networks, such as GPT-4, which may estimate as many as 1.76 trillion parameters [124]). Data types that may include thousands to millions of variables (e.g. genomics, neuroimaging, and digital health [88]) are often referred to as “high dimensional”, given that each variable or data type constitutes a “dimension”, or aspect, of measurement.

**Multilevel** research designs recognize that mental health disorders can be influenced by factors at various levels of organization, ranging from molecular and cellular processes to individual, family, and societal contexts. The term is often used in the context of research on the social determinants of health to describe ecological theories and associated methods in epidemiology and public health [125]. Multilevel is also a statistical term often used to describe mathematical models composed of multiple functions that are embedded within one another (sometimes used interchangeably with the term “hierarchical modeling”) [126]. A prototypical example, the linear mixed model, is a popular technique for analyzing correlated or repeated-measures data, and involves the estimation of both between- and within-group level effects, each approximated by its own model “level”.

**Multi-scale** research designs acknowledge that mental health disorders can be studied at different time scales and spatial resolutions. For example, genetic and epigenetic changes can occur over generations, while neurobiological and psychological processes unfold across different stages of an individual's life. Multi-scale research aims to integrate data across different *temporal and spatial dimensions*, providing insights into the dynamic interplay between factors that contribute to mental health disorders over time and across various levels of organization. Data types that may include thousands to millions of variables (e.g. genomics, neuroimaging, and digital health [88]) are often referred to as “high dimensional”, given that each variable or data type constitutes a “dimension” or aspect of measurement.

#### Genome, phenome, and exposome

Genome, phenome, and exposome are terms used to describe different aspects of an individual's biology and environment, as well as their potential impact on health outcomes, such as mental health disorders.

The **genome** refers to the complete set of genetic information (DNA sequence) of an organism, including all gene coding and non-coding sequences. In the context of neuropsychiatric research, studying the genome can help identify genetic variants associated with mental health disorders, providing insights into the underlying biological mechanisms and potential targets for treatment. Techniques like array-based genotyping and whole-genome sequencing are used to collect data for high-dimensional, multivariate analyses, such as the genome-wide association study (GWAS) [127], that explore the heritable component of neuropsychiatric conditions, including the effects of common and rare forms of genetic variation [128]. Related data types, such as transcriptomics, proteomics, and metabolomics are sometimes referred to broadly as “genomics”, but more accurately can be considered under the broader umbrella term “multi-omics” [129, 130], which includes all genomic-scale molecular measurements including and beyond the sequence of DNA itself.

The **phenome** encompasses the observable traits and characteristics (phenotypes) of an individual, which result from the interaction between their genetic makeup (*genome*) and environmental factors (*exposome*). In neuropsychiatric research, the phenome might include behavioral, cognitive, psychological, and neurological attributes associated with mental health disorders and encompasses both qualitative and quantitative measures. Techniques such as neuroimaging, cognitive testing, and clinical assessments are often used to investigate the phenome in neuropsychiatric research, and initiatives like the Human Phenome Project [131] and large-scale population biobanks [91, 101, 102], have enabled the phenome-wide association study (PheWAS) [132], which essentially extends the concept of GWAS across thousands of measured phenotypes [133].

The **exposome** refers to the totality of environmental exposures an individual encounters throughout their lifetime, from conception onwards [134, 135]. This includes physical, chemical, lifestyle, and psychosocial factors that may impact health outcomes, such as air pollutants, family dynamics, and state or provincial laws. In the context of mental health, studying the exposome helps researchers understand how environmental factors contribute to the development, progression, or resilience of mental health disorders. The epigenome, which describes modifications (e.g. CpG methylation, histone acetylation) to cellular DNA that do not include changes to its nucleotide sequence [136], is often considered a biological proxy of the exposome [137].

## Whole Person Modeling

### The purpose of Whole Person Modeling

In its ideal form, a model of illness should accurately describe the full complement of contributing mechanisms and their consequences, facilitating prediction and intervention. Assuming a biopsychosocial model of mental health, the contributors to illness and well-being are both intrinsic and extrinsic, including inter-individual differences and complex interplay between genome, phenome, and exposome. Given the multi-scale nature of contributing factors to human psychology, such an ideal model would be transdisciplinary and multivariate, and built on several tiers of temporally and spatially-resolved phenomena and systems. Taxonomies to differentiate and organize these tiers are available, for example, in RDoC framework [138], which seeks to describe the discrete and overlapping contributions of genes, molecules, cells, circuits, physiology, behaviors, and environment (including social factors) to specific functional dimensions of behaviour. While a utopian ideal of truly personalized medicine is unlikely to be realized [139], the enterprise of embracing transdisciplinarity in our models of mental health—and explicitly testing their assumptions—is now underway [61, 140]. In defining Whole Person Modeling below we hope to demarcate and operationalize a class of research approaches that adopt this transdisciplinarity and biopsychosocial model of mental illness.

### Defining Whole Person Modeling

We propose the term Whole Person Modeling to refer to an approach to the analysis of data in studies of mental health that meets all three of the following criteria:

A) The analysis is *multivariate and quantitative*, meaning it considers at least one key outcome of interest (most typically a diagnosis, symptom, change in symptoms, or measure of cognitive or social functioning) statistically modeled by two or more additional variables. Qualitative research designs, while invaluable for informing good study design and developing tools for measuring social and behavioural phenomena [141, 142], are not considered Whole Person Modeling here, as they do not include the objective study of biological signals.
B) The analysis is *biopsychosocial*, meaning variables included in the analysis encompass measurements of biological and either psychosocial or other environmental phenomena. For consistency, we consider the measurement of biological phenomena here to be equivalent to the measurement of a “biomarker”, which is defined by the FDA-NIH Biomarker Working Group as “a defined characteristic that is measured as an indicator of normal biological processes, pathogenic processes or responses to an exposure or intervention” [143].
C) The analysis is *transdisciplinary* and *integrative*, meaning that neither data types nor specific variables are considered independently of one another. This means that the study must include models that aggregate, combine, or connect biopsychosocial variables in some way.

Thus, Whole Person Modeling entails the application of existing or novel statistical methods to real-world datasets, including clinical and population-based, usually for the purposes of diagnostic or sub-diagnostic classification, symptom prediction, or treatment response in mental illness. The study design must explicitly model its primary outcome(s) using a multi-domain, biopsychosocial input feature space including at least one objective biological data type (including, but not limited to, neuroimaging, genomics, and/or electrophysiology), and more than one psychological, social, demographic, behavioural, lifestyle, or other environmental measure. Following this, studies testing interactive models but only within a single data domain (e.g., integration of multi-modal neuroimaging metrics for disease classification [144, 145], multi-omic integration [146], and gene–gene interaction models [147, 148]) are not considered Whole Person Modeling. Similarly, studies examining multidisciplinary contributors to diagnosis or prognosis from questionnaires, clinical interviews, or other subjective behavioural assessments exclusively (i.e. not including biological measures) also do not meet our criteria. Some illustrative examples are shown in Table 1.

**Table 1:** Illustrative examples of criteria for Whole Person Modeling

Criteria			Examples
(1)	(2)	(3)	
✕	✕	✕	Description of prevalence of self-reported anhedonia among youth. *(outcome)*
✓	✕	✕	Basic genome-wide association study (GWAS) *(gene 1 → outcome; gene 2 → outcome… gene n → outcome)*
✕	✓	✕	Not possible, as defined
✕	✕	✓	Qualitative study of joint anthropological and sociological perspectives on social networks and mental health in communities. (*environment mode 1 + environment mode 2 → outcome*)
✓	✓	✕	Correlating multiple biomarkers and environmental factors with symptoms independently *(gene → outcome; environment → outcome… K → outcome)*
✓	✕	✓	Multi-omic integration. *(gene* + *protein* + *metabolite* + *methylation → outcome)* Imaging-genetics. *(gene* + *neuroimaging → outcome)*
✕	✓	✓	Not possible, as defined
✓	✓	✓	Whole Person Modeling. (gene + environment + *K* → outcome)

Criteria (1) multivariate & quantitative, (2) biopsychosocial, (3) transdisciplinary & integrative. *K* denotes any number of biopsychosocial data types or variables

Here we note that studies which include chronological age, sex assigned at birth (or defined by sex chromosome dosage), or genetic ancestry (dimensional or categorical) as their sole biological data inputs, but treat them as nuisance covariates, do not qualify as Whole Person Modeling as they do not make a direct assessment of the effects or added predictive value of these factors (e.g. reporting of relevant statistics for models with vs. without covariates). By contrast, studies that explicitly model age, sex, or ancestry and interpret their effects on primary outcomes in combination with at least one objective biomarker would be considered a minimal whole person model. In this scenario, the variables of age, sex, or ancestry can be loosely thought of as “identity context” rather than as a biomarker or modifiable environmental exposure.

We emphasize that while no within-discipline, univariate, bivariate, or unimodal approaches can be considered Whole Person Modeling in and of themselves—these approaches are importantly parallel and antecedent to Whole Person Modeling. Without exploring and decoding complexities within individual domains and disciplines, the exercise of Whole Person Modeling would amount to little more than brute-force, “black-box” algorithms uninformed by the systems they are attempting to model. The entire continuum of approaches, including highly focused, self-contained research programs, is required for successful Whole Person Modeling, as data and knowledge are funneled forward toward integration through active communication and collaboration.

We also note that our concept of Whole Person Modeling is tightly linked to the “transdisciplinary holistic integrative psychiatry” approach proposed by Jakovljevic and Jakovljevic [61, 149]. Our description of Whole Person Modeling is intended to provide an operational definition and accessible guide—specifically for students and researchers working with data—that compliments their theoretical groundwork on the “body–mind–energy–spirit holodigm” and the pursuit of predictive, preventive, precision, person-centered, and participatory medicine (5PMed) in psychiatry (see [61]).

### Degrees and “flavors” of Whole Person Modeling

Given the aforementioned criteria, it is possible to place Whole Person Modeling studies on both a quantitative spectrum (i.e. how many data types; how many variables or features; model complexity) and within a qualitative categorization (i.e. “flavors” of hypothesis-generating vs. hypothesis-driven studies).

#### The spectrum of data breadth and depth

On one end of the quantitative spectrum, a Whole Person Modeling study may include relatively few variables or data types and involve a relatively straightforward analysis. An illustrative example (and perhaps the most common type of Whole Person Modeling study) is the gene-by-environment (G × E) interaction study [150–152], in which genetic variants or composite risk scores (e.g. polygenic risk scores) are tested for effects on an outcome that are dependent on a third environmental influence or exposure. This kind of study can be hypothesis-generating (e.g. a genome-wide G × E scan [153–155]) or hypothesis driven (a candidate gene and mechanism [156]). In the latter case, a study may be as simple as analyzing a single outcome, single genetic variant, and single environmental exposure. A famous and enduring study of this type found that a serotonin transporter (5-HTT) gene promoter polymorphism moderated the effect of stressful life events on depression [157, 158]. This design can be thought of as the simplest kind of whole person model as we have defined it above: a single outcome (in this case, a clinical variable) is being modeled as the product of one biological factor and one psychosocial factor (potentially in the presence of additional covariates). Of course, more complex models are encouraged and ultimately will be necessary for providing more nuanced explanations of mental illness.

On the opposite end of the data breadth and depth spectrum are complex, multilevel machine learning or network-based analyses of many biopsychosocial domains (hundreds or even thousands of variables), which permit unconstrained nonlinear and interactive effects. For example, Koutsouleris et al. [159] analyzed multisite, longitudinal data (from the Personalised Prognostic Tool for Early Psychosis Management (PRONIA) study [160]) to predict psychosis in individuals at clinical high risk. Their comprehensive approach included the sequential building of a combined prognostic model combining machine learning-derived algorithms and human expert-based models (following principles of expert-augmented ﻿﻿machine learning﻿﻿ [161]), which ultimately was able to achieve 86% predictive accuracy. Different iterations of their model included 141 clinical-neurocognitive measures, human expert-based predictions, 10 polygenic risk scores for schizophrenia, and whole brain maps of gray matter from MRI.

Other examples of complex, high-dimensional, and multi-modal Whole Person Modeling designs can be found in the study of Alzheimer’s disease; Singanamalli et al. (2017) [162] used cascaded multi-view canonical correlation analysis to classify stages of Alzheimer’s disease in ADNI [163], integrating cognitive (1 feature), structural MRI (327 features), fluorodeoxyglucose (FDG)-positron emission tomography (PET) (2 features), cerebrospinal fluid (CSF) proteomics (3 features), plasma proteomics (146 features), and *APOE* genotype data. They found that different combinations of input features performed better for classifying different stages of Alzheimer’s disease than either the combination of all data modalities or any single modality alone. Since ADNI pioneered the model of highly multidisciplinary, multimodal, longitudinal data collection and global data sharing nearly 20 years ago, it is commonly used for Whole Person Modeling [164–172].

#### Hypothesis-Generating Whole Person Modeling

The primary goal of hypothesis-generating Whole Person Modeling is to identify and prioritize variables that are most strongly associated with a key outcome of interest. This flavor of Whole Person Modeling may also be favorable in situations where there are no existing theoretical models for the nature of relationships between data types under study. This approach can also be thought of as “hypothesis-free” or “exploratory” in nature—terms which are hotly debated (see [173] and [174]). Knowledge resulting from this approach can support the design of new observational and interventional studies that better balance the cost–benefit of measurement burden vs. information collected. It can also be used to inform decision makers and improve public health initiatives at the population scale (e.g. “precision public health” [175]).

A hypothesis-generating Whole Person Modeling approach at minimum involves four steps: (1) identifying a primary outcome of interest, (2) selecting or collecting a human cohort dataset of sufficient size with genomic, phenotypic, and/or exposomic characterization, (3) identifying or constructing biopsychosocial variables (features) for modeling, (4) fitting and validating one or more multivariate model(s) to the outcome and (5) explaining or interpreting the model(s). One example of such a design is that by Spechler et al. (2018), who sought to understand the predictors of cannabis use in youth: (1) Their outcome of interest was a binary variable representing yes or no to using cannabis between baseline and two-year study follow-up; (2) their dataset was the IMAGEN study [176] (n = 1,581, age 14 at baseline); (3) their biopsychosocial variables (total of 2,413) included self-reports of demographics, personality, life events, and parental drug use; cognitive performance and intelligence scores; genetic data for 108 candidate genetic variants and one polygenic risk score; and 278 variables each from functional MRI task-based maps and structural MRI grey matter volume maps; (4) They fit these features to their outcome using regularized logistic regression (elastic net [177]) with tenfold cross-validation. They also modeled their outcome separately for male and female groups; (5) They interpreted their models by (a) reporting variables selected by the elastic net procedure and (b) by hierarchically testing models and measuring model fit after the inclusion of each domain-specific set of predictors. In support of the Whole Person Modeling approach, they found that models containing both psychosocial and sex-specific brain features performed best, generating new hypotheses on the constellation of roles played by novelty-seeking personality traits, opioid receptor gene variants, medial frontal and cerebellar cortical development, and biological sex in cannabis use.

Importantly, although Whole Person Modeling starts from a biopsychosocial approach to illness, it does not restrict hypothesis-generating studies from challenging it. For example, Dinga et al. [178] used machine learning to analyze unipolar depression in the Netherlands Study of Depression and Anxiety (NESDA) [179], combining demographic, psychological, clinical, and biological data (including biomarkers of hypothalamic–pituitary–adrenal axis, inflammation, metabolic markers, autonomic nervous system, vitamin D, and neuronal growth factors). They achieved a balanced accuracy of 66% for prediction of depression at two-year follow-up, but found that only self-reported baseline depressive symptom severity was a significant predictor of future risk; no benefit was seen with the inclusion of other domains of data. Similarly, McNamara et al. [180] used sociodemographics, polygenic scores, resting electroencephalography (EEG), pupillometry, actigraphy, and cognitive task data (up to 139 features) to classify depression in a sample of 217 adults. Cross-validated model performance varied substantially across outcomes (i.e. anhedonia, general distress, and lifetime history of depression), and they too found that biological measures added very little to their model’s predictive value.

#### Hypothesis-Driven Whole Person Modeling

The primary goal of hypothesis-driven Whole Person Modeling is to explicitly test the assumptions of a theoretical model of illness risk or progression. Candidate G × E interaction studies broadly fall under this category but to illustrate other possibilities for hypothesis-driven Whole Person Modeling designs, we have selected three additional examples:

1. Veldsman et al. [181] sought to better understand the complex relationships between cerebrovascular risk factors, brain health, and cognition. They developed a conceptual hypothesis of the role of the frontoparietal network in the relationship between age, cardiovascular disease risk factors (a composite of *APOE* genotype, blood pressure, diabetes, and smoking), white matter hyperintensities (measured with MRI), and executive function, and then modeled it explicitly using structural equation modeling (SEM). They fit their model using data for 22,059 participants from the UK Biobank and found support for the idea that targeted and time-sensitive control of blood pressure in mid-late life may be most beneficial to both cognitive and brain health.
2. Complex links exist between depressive, neurodegenerative, and metabolic disorders [182]. Using data for 11,355 participants from the Brazilian Longitudinal Study of Adult Health (ELSA-Brazil) [183], Duinkerken et al. (2020) used multivariate logistic regression to selectively model several domains (what they refer to as “blocks”) of data, grouped specifically according to their theoretical proximity to a current depressive episode (including classical predictors of depression, e.g. age, sex, race, and education; psychosocial factors, e.g. income, marital status, life events, and experiences of discrimination; cardiovascular factors, e.g. alcohol consumption, smoking, lipid-lowering medications, BMI, and blood lipids; and other liver enzymes, urinary albumin to creatinine ratio, C-reactive protein (CRP) and insulin resistance). In their model, metabolic factors such as glycemic control were not independently related to a current depressive episode, providing evidence for a biopsychosocial approach to treating patients with metabolic abnormalities who may be at elevated risk for depression.
3. Patients with post-stroke depression have lower levels of brain-derived neurotrophic factor (BDNF), which may be due to a pre-existing vulnerability or ischemic damage induced during stroke [184]. The effect of these changes on risk for post-stroke anxiety or depression may be exacerbated by social factors, leading to changes in the hypothalamic–pituitary–adrenal (HPA) axis that can perpetuate further BDNF decline. This aligns with biological impairment theories (i.e., the neurotrophin hypothesis of depression) and psychosocial vulnerability theory, both of which coexist in stroke patients. Han et al. [185] tested this model integrating 198 features (146 proteomic, 22 sociodemographic and 30 clinical) with regularized regression (LASSO [186]), finding that the combination of divorce/separation and BDNF levels had higher predictive accuracy for post-stroke depression than either factor alone, which was further increased by a history of smoking.

### Methods for Whole Person Modeling

#### Multivariate statistics

While a full survey of multivariate statistical methods appropriate for Whole Person Modeling is beyond the scope of this review, here we outline some common approaches, providing examples and resources for the interested learner.

Perhaps the most common statistical models used in multivariate data-driven mental health research are ordinary least squares (OLS) linear regression and logistic regression [187]. These methods model continuous and binary outcomes, respectively, as the linear combination of independent variables, and include assumptions that observations (e.g. study participants) are independent (i.e. they are not clustered, as in longitudinal data analysis) and that outcomes are generally normally distributed (i.e. the assumption is that regression model residuals should follow a normal, or Z, distribution) [188]. They are simple examples of a broader class of generalized linear models which are more flexible to different data distributions and assumptions. Multiple regression also serves as the foundation for basic statistical mediation analysis, described by Judd and Kenny (1981) [189], which essentially involves fitting a sequence of three regression models with independent (*X*), dependent (*Y*), and hypothesized mediator (*M*) variables, and contrasting their parameter estimates to test for the portion of *X* → *Y* effects that are mediated by *M*; *X* → *M* → *Y* (e.g. using the Sobel test [190]).

An example of Whole Person Modeling making use of multiple regression and statistical mediation is the analysis of biopsychosocial mediators of the effects of physical activity on psychiatric symptoms in youth. Rodriguez-Ayllon et al. [191] analyzed 4,216 Dutch children at three time points (at age 6,10, and 13), correlating externalizing and internalizing behaviors with levels of physical activity, and then testing regression-based mediation effects of brain volume, white matter microstructure, resting-state connectivity, self-esteem, body image, and friendship, where significant correlations were observed. They found that self-esteem mediated the association between sports participation and internalizing symptoms. Despite their relative simplicity and interpretability, linear and logistic regression can be used to model fairly complex phenomena, including terms to represent non-linear and interactive (i.e. multiplicative) effects. For authoritative resources on regression modeling, see Harrell’s online portal [192].

To accommodate the very high dimensional nature of some Whole Person Modeling designs, latent space models and dimensionality reduction techniques can be useful tools (e.g. canonical correlation analysis (CCA), principle components analysis (PCA), and partial least squares regression (PLS)). These techniques are means of collapsing, selecting, or combining multiple variables to arrive at a smaller set of (ideally) more informative features. This is often an initial step in machine learning analytical pipelines [193], but is also a feature in non machine learning-based Whole Person Modeling work as well [194–196]. Given the natural organization of biological and social systems as networks [197], statistical network-based methods are also often used to handle complex data in biopsychosocial research [198]. For example, Gamberger et al. [199] developed a novel multi-layer clustering method to differentiate trajectories of cognitive decliners in ADNI based on 43 longitudinal and cross-sectional features (including genotype, structural MRI, PET, CSF, cognitive, and educational data types).

In higher dimensional applications, Similarity Network Fusion (SNF) [200] and its derivatives [201, 202] are network-based, hypothesis-generating clustering methods specifically designed to integrate variables from multiple data modalities at a multi-omic scale. While most often used for integration of data types within a single discipline (e.g. multi-omics), SNF has been used to identify subtypes of psychiatric illness in youth using combinations of over 130 measures from multi-modal structural MRI, neurocognitive tests, behavioural assessments, and sociodemographic questionnaires [203, 204].

#### Machine learning and artificial intelligence

Machine learning refers very broadly to a process in which algorithms (typically statistical models) are automatically developed (rather than designed explicitly) to accomplish some task (e.g. prediction or classification) through the use of data from prior experience [205]. As models become highly complex, the ability of these algorithms to perform pattern recognition tasks increases impressively. This is an appealing set of qualities for integrative, biopsychosocial modeling (particularly of the hypothesis-generating flavor) where the structure of complex relationships between many variables is not known a priori*.*

While machine learning is built on multivariate statistical methods, it can be conceptualized as distinct from the field of statistics; for example, Bzdok et al. [206] posit that “statistics draws population inferences from a sample, and machine learning finds generalizable predictive patterns”. However, as they also note, this conceptualization can lead to the false views that prediction and inference are mutually exclusive; often, the same model can be used for both purposes, and there is no reason to assume that predictive models should not be interpretable or offer opportunities for inference. It is also worth pointing out stereotypically “statistical” models, such as linear regression, are also used within a machine learning framework and qualify as such. While they are not the same, the true nature of the distinction between machine learning and statistics is actively debated [207].

The term Artificial intelligence (AI) has no widely accepted definition, beyond that it involves some degree of attempt to replicate or exceed human-level capability in performance of a specific task (narrow AI) or in general (general AI). In mental health, AI research remains in its infancy, though visions for how AI may overcome common issues related to sample size, model construction, evaluation practice, and the very concept of mental disorders have been proposed [208]. While machine learning is technically a subfield of AI [205], we do caution against using the term AI indiscriminately to describe any machine learning or algorithmic model in the context of research, as it can provoke unwarranted negative reactions due to evolving public concerns and mistrust in the development of AI-based systems and technologies [209, 210].

In Whole Person Modeling, machine learning is frequently used for its ability to handle complex, high dimensional data. Several examples of Whole Person Modeling Studies using regularized regression are described in Sect. 3.3.2. Other models are also used, for example Lalousis et al. [211] used support vector machines to build two models of depression and psychosis in recent-onset patients with two sets of variables: 151 features from clinical and neurocognitive tests including anhedonia, social functioning and cognition deficits, and brain-wide maps of gray matter volume from MRI. They then compared each model to the combined (via stacking [212]) model, finding that different types of both social and physical anhedonia were important in the classification of both psychosis and depression, and that the influence of gray matter volume was dependent on the clinical group.

Clustering can also be accomplished in machine learning-based workflows. An example is a recent Whole Person Modeling study by Allesøe et al. [213] who used variational autoencoders, a type of artificial neural network (see Krogh [214] for an accessible primer), to cluster and subtype 19,636 individual with major depressive disorder and/or schizophrenia from a large Danish population-based case-cohort sample (the Integrative Psychiatric Research Consortium (iPSYCH) [215]). Their model integrated information ranging from disorder severity, history of mental disorders and disease comorbidity, genetics, and medical birth data.

Machine learning is also useful for Whole Person Modeling using simulation-based methods. Fisher et al. [216] created a synthetic longitudinal dataset, using a machine learning method called the conditional restricted Boltzmann machine (CBRM), based on to data from the Coalition Against Major Diseases (CAMD) Online Data Repository for Alzheimer’s disease (CODR-AD; n = 1,909) [217]. Their model captured the 18-month evolution of each sub-component of cognitive scores (specifically from ADAS-Cog and MMSE scales), blood laboratory tests, and their associations with baseline clinical characteristics (a total of 44 features). This is an example of the “digital twin” approach, which has been accelerated by machine learning methods and fundamentally involves the simulation of a virtual entity (or model) that represents as faithfully as possible a physical object or system (such as a human) [218]. In mental health research, this approach may allow for virtual testing of different treatments, obviating long, expensive trial-and-error processes, and benefiting areas of monitoring, diagnostics, prognostics, and treatment guidance without any additional human participant burden [219].

Despite their complexity and associated learning curves, machine learning approaches have several distinct advantages over simpler models: for example, multivariate pattern recognition paired with local interpretability measures, such as SHapely Additive exPlanations (SHAP) values [220], allows for model inference at the individual rather than group level. This can provide crucial insight when the goal of a study is to understand how different groups or strata within a dataset differ with respect to the influence of clinically important predictors (as in precision psychiatry [221]). Machine learning can also be advantageous over multivariate models such as logistic regression as they can require fewer variables to achieve better estimates and handle correlated data well, which reduces the loss of power that comes from correction for multiple comparisons.

For comprehensive reviews on machine learning and deep learning in mental health research, see [222–225]. Excellent resources, including primers an practical tutorials for students and researchers interested in approaching machine learning research and explainable methods are widely available (we highly recommend [226]). Popular software packages in R and Python programming languages, such as scikit-learn (Python) [227] and Classification And REgression Training (caret) [228], have made these methods accessible to the non-specialist.

## Challenges in Whole Person Modeling

### Integration for the sake of integration

Despite the prominent optimism for transdisciplinary approaches outlined in this review, and the rise of interdisciplinary research (especially for health-related fields) [229], this optimism is not universally shared. Jacobs [230] and others provide thoughtful critiques of the widely held assumptions about the ubiquitous benefits of transdiciplinarity, defending the concepts of specialization and the traditional organization of higher education and research institutions (which have been met with rebuttal [231]). The sentiment that more is always better, e.g. larger datasets, more variables, is also not unanimous. Schofield and Das-Munshi [232] suggest that reckless adoption of a “big data” mentality in mental health research can lead scientists to discard important theory, experience overconfidence in predictive models (“big data hubris”, see [233]), and miss important details related to data validity and replicability. While greater size and complexity may not always better for research, it is sometimes the case that even small increases in model complexity can meaningfully improve issues related to bias and confounding. For example, Keller [234] demonstrated that the inclusion of covariate × environment and covariate × gene interaction terms can successfully overcome model misspecification in regression testing of G × E interactions.

### Model specification, overfitting, under-fitting, and sample size

Another major challenge in Whole Person Modeling is mitigating risks related to model specification (i.e. “how complex should a model be?”) which are shared by all quantitative statistical modeling approaches. Machine learning studies in particular are prone to suffering from overfitting (having low bias and high variance), in which a model is effectively “over-trained” on one dataset, resulting in poor generalized task performance (e.g. prediction or classification) in another dataset. Conversely, models can also be under-fit (or “underspecified”—having high bias and low variance) [235], resulting in misleading parameter estimates and misattribution of variable importance. Lever et al. [236] review these concepts accessibly, including the bias-variance trade-off and model selection.

Related to the challenge of model specification is that of appreciating the statistical limits of a given dataset and the relationship between the number of variables included in an analysis and the number of independent observations. As model complexity increases, so does the need for more independent observations to mitigate the chances of overfitting. For example, it has been shown that adequate sample sizes (n > 1000) are critical for reproducible discovery in high-dimensional analyses of brain-wide neuroimaging studies in mental health [237]. The rapid adoption of high dimensional machine learning techniques in the absence of appropriately large datasets has led to a reproducibility “crisis” in biomedical research [238], and machine learning studies in psychiatry show a systematic bias toward better model performance as sample size decreases [239]. Despite this, the sample size debate remains active, and some have raised concerns over a pervasive and misguided preoccupation with sample size, labeling it as false dogma and blaming it in part for the lack of innovation and translation in medical research [240, 241]. Whole Person Modeling studies, as defined here, are not bound by the requirement for large sample sizes, so long as the design is chosen appropriately. Volovici et al. [242] provide a comprehensive overview of common mistakes in the use of machine learning in clinical research and how to avoid them.

### Lack of gender, ethnic, and ancestral diversity

Unfortunately, biopsychosocial research, especially in large cohort datasets, has been historically marred by lack of foresight when designing assessments of identity, including gender and sociocultural ethnicity. This is a critical misstep, since the substantial impact of these factors and their complex intersections on well-being have long been known and should not be ignored [243]. Some studies are now beginning to correct this. For example, the ABCD study, which is not specifically focused on the study of gender or sexuality, created a Gender Identity and Sexual Health working group which selected study instruments with documented reliability and validity, repeatability, developmental sensitivity, minimal burden, and non-pathologizing and non-stigmatizing language [244]. Major funding organizations are recognizing the importance of this approach. For example, CIHR now requires all researchers applying for funding to gain accreditation in Sex and Gender-Based Analysis Plus (SGBA +) [245].

In the search for mechanistic explanations of mental illness, mounting evidence suggests that confidence in the axiom that minimizing heterogeneity in study design is universally beneficial may be misplaced. A key example of this is in psychiatric genomics—a core element of precision medicine paradigms and commonly used biological data type in Whole Person Modeling—where the inclusion of ancestrally diverse and admixed populations is now recognized to benefit discovery rather than hinder it [246–249].

Recognition of the importance of diversity can also have a profound impact on global educational initiatives. Martin et al. [250] outline their experiences building interdisciplinary research and capacity-building programs (the NeuroGAP-Psychosis research study [251] and the Global Initiative for Neuropsychiatric Genetics Education in Research (GINGER) training program) that prioritize equity and span institutions in Ethiopia, Kenya, South Africa, Uganda, and the United States.

### The challenges of organization, sharing, and harmonization

The primary practical challenge for Whole Person Modeling is no doubt the difficulty of accessing, organizing, and curating data. While datasets are becoming increasingly available in the technical sense, the institutional and governmental procedures and legal frameworks that constitute data governance remain underdeveloped. As a consequence, the processes for gaining access to and transferring data (even between directly collaborating institutions) are often surprisingly arduous. Additional barriers to sharing include lack of informed consent in data collection, lack of ethics approval for data sharing, lost data, or researcher turnover [252]. Piasecki and Cheah [253] review some other perceived barriers in depth, with a focus on how access to data and data ownership are intertwined with considerations of ethics and risk mitigation, among others. Models of data access are still varied and can be quite complex. To get around this, some have advocated for fully open sharing models [254], in part to reward the altruistic efforts of study participants (in particular those who participate in clinical trials), though this model does not address other important issues related to governance.

While widely accepted as important for progress in modern mental health research, data sharing across groups, institutions, or political borders does not always occur. For example, the All of Us Study does not currently have a roadmap for sharing of data outside of US-affiliated institutions, and a striking majority of researchers do not share their data when requested [255], despite published data availability statements. Data sharing also requires the detailed maintenance of provenance (the tracking of origins and manipulations of data as they move) and documentation, without which reproducibility and translatability suffer irreparably. The Findable, Accessible, Interoperable, Reusable (FAIR) principles offer a framework for organizing and sharing data in a responsible way [256].

Harmonization refers to the process of transforming or reshaping data from two different sources such that they can be analyzed together [257, 258]. This is clearly a major hurdle for Whole Person Modeling, where large, complex, multi-site datasets are required to achieve feature breadth and depth. The aforementioned Maelstrom Research Project and NIMH Data Archive (NDA) are examples of data catalogs and repositories that include data schemas for contributing researchers to adopt for ease of harmonization. The AMP-AD initiative has also received specific funding from the NIH for harmonization of measures within its component cohorts [259].

### Building a transdisciplinary career

Finally, the disciplinary culture of academia (see Sect. 2) has produced barriers for trainees, early career investigators, and even established opinion leaders who wish to pursue or transition to careers in highly transdisciplinary research programs. Sellberg et al. [260] articulate the challenges well, touching on three main issues: (1) Inadequate institutional support. Despite vocal support for transdisciplinary research, it is rarely well understood, supported, or valued at the institutional level. There are unique burgeoning demands of transdisciplinary research which are exacerbated by a lack of appropriate metrics of success, since standard academic metrics largely focus on measuring the number and impact (or value) of publications and research grants. These can be more challenging to achieve since highly transdisciplinary work does not fit within current organizational structures of peer review. (2) Time and resource constraints. Transdisciplinary research often results in increased research transaction costs, leading to time and resource constraints for those involved. The time and resources (social, personal, and research-related) required to achieve 'good' transdisciplinary research (meaning research that is both societally impactful and respectful, and scientifically rigorous) are immense and often surpass those of traditionally hyperfocused research. (3) Well-being and stress management. Transdisciplinary researchers (trainees in particular) may experience heightened anxiety and stress related to the risk of falling behind in their research career, building strong core skills while avoiding becoming a “jack of all trades, master of none”. Nash and colleagues [261, 262] have also explored these challenges in depth.

## Recommendations for Whole Person Modeling

### Four core principles for successful Whole Person Modeling

(1) Work with others: making the effort to be informed of and collaborate with disciplines, people, and ideas other than those you are most familiar with. Diversity, in its many forms, drives innovation [263–265]. (2) Actively seek formal and informal programs, seminars, courses, and tutorials on the use of multivariate, multiscale statistics. Prioritize fundamental concepts of data distributions, missingness, resampling, measurement error, interpretation, and reporting. (3) Whenever possible, explicitly test the assumptions that an increased variety of data, depth of data, or model complexity is (a) enhancing predictive performance, generalizability, or interpretability of the model, or (b) providing novel mechanistic insights. (4) Consider the experience of the individual from both illness and wellness perspectives. An often overlooked fact in mental health research is that wellness is not merely defined as the absence of illness; this is articulated by the definition of health in the Constitution of the World Health Organization (1946):

*“Health is a state of complete physical, mental and social well-being and not merely the absence of disease or infirmity.” *[266]

This is important given that we now have the data resources capable of probing the distinction between illness and wellness. Mead et al. [267] review the paucity of biopsychosocial literature on mental wellness, and suggest a multi-level framework for interrogating it.

### Engaging clinician and participant stakeholders in study design

When performing Whole Person Modeling, especially with the goal of deriving usable knowledge or impacting clinical practice, researchers should foster a culture of reference to both expertise and experience. Stakeholder engagement at the level of research study or more broadly for policy development [268] is essential for understanding and achieving the goals of transdisciplinary science. As such, inclusion of individuals with lived experience to inform study design, analytics, and implementation throughout the project lifecycle is increasingly encouraged (as by the CIHR Strategy for Patient-Oriented Research (SPOR) program [269]) [270]. This is reinforced explicitly by the WHO Mental Health Action Plan: “Persons with mental disorders and psychosocial disabilities should be empowered and involved in mental health advocacy, policy, planning, legislation, service provision, monitoring, research and evaluation.” [271]. Engagement with members of the population being served by research is especially important in studies of youth mental health, where ongoing consultation and dialogue can improve the safety and comfort of participants, enhance the trustworthiness of findings, and diversity modes of scientific dissemination, among other benefits [272]. Ethical considerations cannot be taken lightly here: integrative mental health research involving sensitive data types requires linkage of potentially identifiable information, and the deployment of tools built on these data should be rigorously tested for fairness to avoid the exacerbation of existing health inequities [273]. In the design of data collection efforts in mental health, resources should be allocated to assessing opinions and attitudes toward the risks and utility of research as it is being conducted [274].

### Creating transdisciplinary educational opportunities

Research mentorship and educational programs adopting the principals and ideas of Whole Person Modeling are electing to telegraph their transdisciplinarity and accept it as integral to their identity. In doing so, they are exposing trainees and collaborators to a range of ideas, methods, and concepts and most will not encounter in traditional career paths. Encouraging this breadth of exposure while maintaining the levels of focus required for competence at each level of education (i.e. undergraduate, master's, doctoral, postdoctoral) will be the major challenge. We believe that the balance between specialization (which is required for high quality science) and transdisciplinarity is not a zero-sum game, and that there is a great opportunity to clarify epistemology for students when artificial disciplinary barriers are dissolved (for example, between neurology and psychiatry [275]). Wall and Shankar [276] anecdotally suggest that keys to transdisciplinary training at the graduate level may be readiness, relationships (among fellow trainees), and being provided with physical, administrative, financial, and social supports.

One way to achieve breadth without sacrificing depth may be through the establishment of core competencies across multiple disciplines, e.g. statistics, scientific computing, psychometrics, and genomics. Another way may be through the pairing of periodical low-throughput, high-attention, transdisciplinary educational initiatives with longer-term, self-guided online learning. There has recently been an explosion in the number of freely available or paid online courses reaching across most conceivable biopsychosocial disciplines and their methods, with increases in enrollment mirroring the transition to virtual learning compelled by the COVID-19 pandemic [277]. However, students may lack the guidance from more senior experts with transdisciplinary experience that is necessary for charting an optimal personalized path through the dizzying array of tutorials and courses. Developing such paths, or personalized learning plans, will be one way for students to gain a foothold in Whole Person Modeling moving ahead, without needlessly reinventing the wheel by creating bespoke curricula from scratch.

## Conclusions

Whole Person Modeling is an emerging field of research, built on transdisciplinary collaboration, multivariate statistics, and embracing the biopsychosocial complexity of mental health. It has been enabled by decades of global investment in large-scale, multi-modal data collection in population-level and clinical studies of health, as well as developments in high-dimensional statistical modeling techniques and a growing culture of open science. If rigorously conducted, Whole Person Modeling studies have the potential to provide a better understanding of multilevel phenomena, deliver more accurate diagnostic and prognostic tests to aid in clinical decision making, and test long standing theoretical models of mental illness and wellbeing. Some current barriers to progress for Whole Person Modeling include challenges with interdisciplinary communication and collaboration, systemic cultural barriers to transdisciplinary career paths, technical challenges in model specification, bias, and data harmonization, and gaps in transdisciplinary educational programs.

## Data Availability

Not applicable.
